# Self-assembly of a strapped linear porphyrin oligomer on HOPG

**DOI:** 10.1038/s41598-021-99881-x

**Published:** 2021-10-14

**Authors:** Abigail Bellamy-Carter, Cécile Roche, Harry L. Anderson, Alex Saywell

**Affiliations:** 1 School of Physics and Astronomy, University of Nottingham, Nottingham, NG7 2RD UK; 2 Department of Chemistry, University of Oxford, Oxford, OX1 3TA UK

**Keywords:** Scanning probe microscopy, Surface assembly, Surfaces, interfaces and thin films

## Abstract

Polymeric structures based on porphyrin units exhibit a range of complex properties, such as nanoscale charge transport and quantum interference effects, and have the potential to act as biomimetic materials for light-harvesting and catalysis. These functionalities are based upon the characteristics of the porphyrin monomers, but are also emergent properties of the extended polymer system. Incorporation of these properties within solid-state devices requires transfer of the polymers to a supporting substrate, and may require a high-degree of lateral order. Here we show that highly ordered self-assembled structures can be formed via a simple solution deposition protocol; for a strapped linear porphyrin oligomer adsorbed on a highly oriented pyrolytic graphite (HOPG) substrate. Two distinct molecule–molecule interactions are observed to drive the formation of two molecular phases (‘Interdigitated’ and ‘Bridge-stabilised’) characterised by scanning tunnelling microscopy, providing information on the unit cell dimensions and self-assembled structure. The concentration dependence of these phases is investigated, and we conclude that the bridge-stabilised phase is a thermodynamically stable structure at room temperature.

## Introduction

Porphyrins are macrocyclic compounds that exhibit a wide range of optical, magnetic, and electronic properties. These properties are intrinsic to the specific porphyrin species under study, and are related to the structure of the macrocycle itself, the formation of metal complexes (metalloporphyrin), and contributions from moieties present at the periphery of the porphyrin^1,2^. The inclusion of additional chemical functionality, in the form of pendant groups, is a promising route towards developing bespoke functionalities^3^. Incorporating these properties into devices, which is likely to require a supporting substrate, has motivated the study of porphyrin-surface systems^4^. Investigation of such molecule–substrate systems is often carried out under ultra-high vacuum (UHV) conditions, facilitating contaminant free, highly controllable, fabrication procedures as well as providing access to characterisation on the single-molecule level via scanning probe microscopy techniques^5^.

The covalent coupling of porphyrins to form oligomer, polymers and macrocycles can result in more complex functionality, with the potential for nanoscale charge transport^6^, light harvesting^7,8^, and quantum interference^9^. However, the size, and thermally labile nature, of these molecules prohibits the use of standard UHV sublimation protocols. A range of alternative deposition techniques have been developed^10^, and we have demonstrated that porphyrin oligomers, polymers and macrocyclic systems can be deposited via an electrospray deposition technique^11–13^. In addition, porphyrin polymers may be formed by using a range of on-surface synthesis techniques^14–17^, including Glaser coupling of porphyrin monomers^18^.

**Figure 1:**
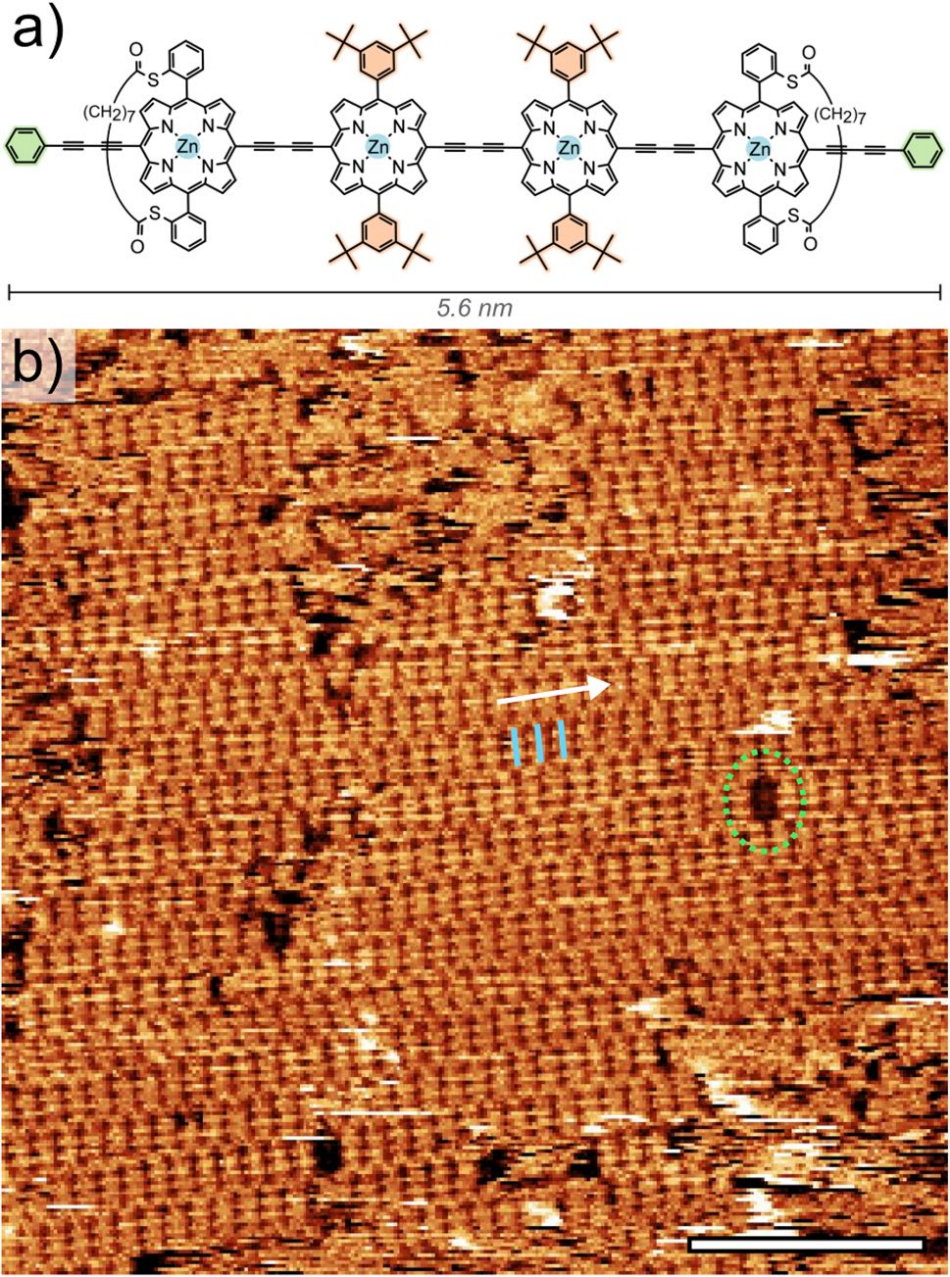
**(a)** The chemical structure of **S4-l-P4. (b)** An overview STM image of a 3.0 μM drop-cast sample showing large, ordered structure assigned to close-packed **S4-l-P4** molecules: ‘Row’ direction indicated with a white arrow, position of three individual molecules shown as blue lines, and a defect assigned to the absence of a single molecule highlighted by a dashed green ellipse. Scale bar is 20 nm.

The porphyrin units in the system under study here are functionalised with bridging alkyl chains (similar to those previously included in a 6-unit porphyrin nanoring^9^). Bridged, or strapped, porphyrins have been extensively studied, and have been proposed as biomimetic analogues to heme^19^ and cytochrome^20,21^ systems, as well as stereoselective catalysts ^22^, and as a route towards the formation of mechanically-locked structures^23,24^. However, the addition of these bridge groups has the potential to alter the nature of the molecular-molecular interactions leading to a lack of long-range order within surface-supported molecular arrays; unless additional groups are added to anchor the molecules^25^, leading to further complications for preparation under UHV conditions. An alternative approach is the solution-based formation of ordered molecular arrays, thereby removing some of the complexities present as part of UHV fabrication. The ordering of such molecular assemblies in solution can be driven by a range of molecule–molecule and molecule–substrate interactions^26–28^; including van der Waals and hydrogen-bonded mediated arrangements. Here we study a four-unit butadiyne-linked porphyrin oligomer, **S4-l-P4** (see Fig. 1a), which was originally designed so that the thio-ester links would cleave and all four sulfur atoms could anchor the oligomer to a gold surface^9^. Here we characterise the adsorption geometry and long-range structures formed by this molecule on the highly oriented pyrolytic graphite (HOPG) surface, with reference to the inter-molecular interactions which govern the observed arrangements. Two distinct molecular arrangements are observed, each stabilised by an alternative set of intermolecular interactions. We determine a weak concentration dependence upon the prevalence of the two structures, and via the tip-induced phase-change between the two structures are able to infer details of the relative stability of the two co-existing structures.

## Results

Close-packed arrays of **S4-l-P4** were observed to form following deposition of the porphyrin oligomer from a toluene solution (3.0 μM) drop-cast on to a HOPG surface (Fig. 1b). The resultant structures are characterised using a scanning tunnelling microscope (STM) with the tip-sample junction within nonanoic acid [1 V bias (STM tip), current set-point 50 pA]. The structure of **S4-l-P4**, shown in Fig. 1a, consists of four zinc porphyrins linked by butadiyne units at the 10,20-*meso* positions. The two central porphyrins are functionalised with 3,5-di-*tert*-butylphenyl groups (di-*t*BuPh) at the 5,15-*meso* positions (indicated in orange in Fig. 1a). The porphyrin units at either end of the oligomer are functionalised at the 5,15-*meso* positions with thioester-linked alkane straps, and the chain is terminated with phenyl butadiyne groups (indicated in green in Fig. 1a).

From the overview STM shown in Fig. 1b several features can be discerned: (i) the molecules within the ordered domain exhibit a preferential ‘row’ direction (white arrow); (ii) the position of individual molecules can be understood as short ‘rods’ arranged perpendicular to the row direction (blue lines); (iii) the assignment of the molecular positions are supported by defects within the ordered domain (dashed ellipse) where it appears that a single molecule is absent from the domain.

**Figure 2:**
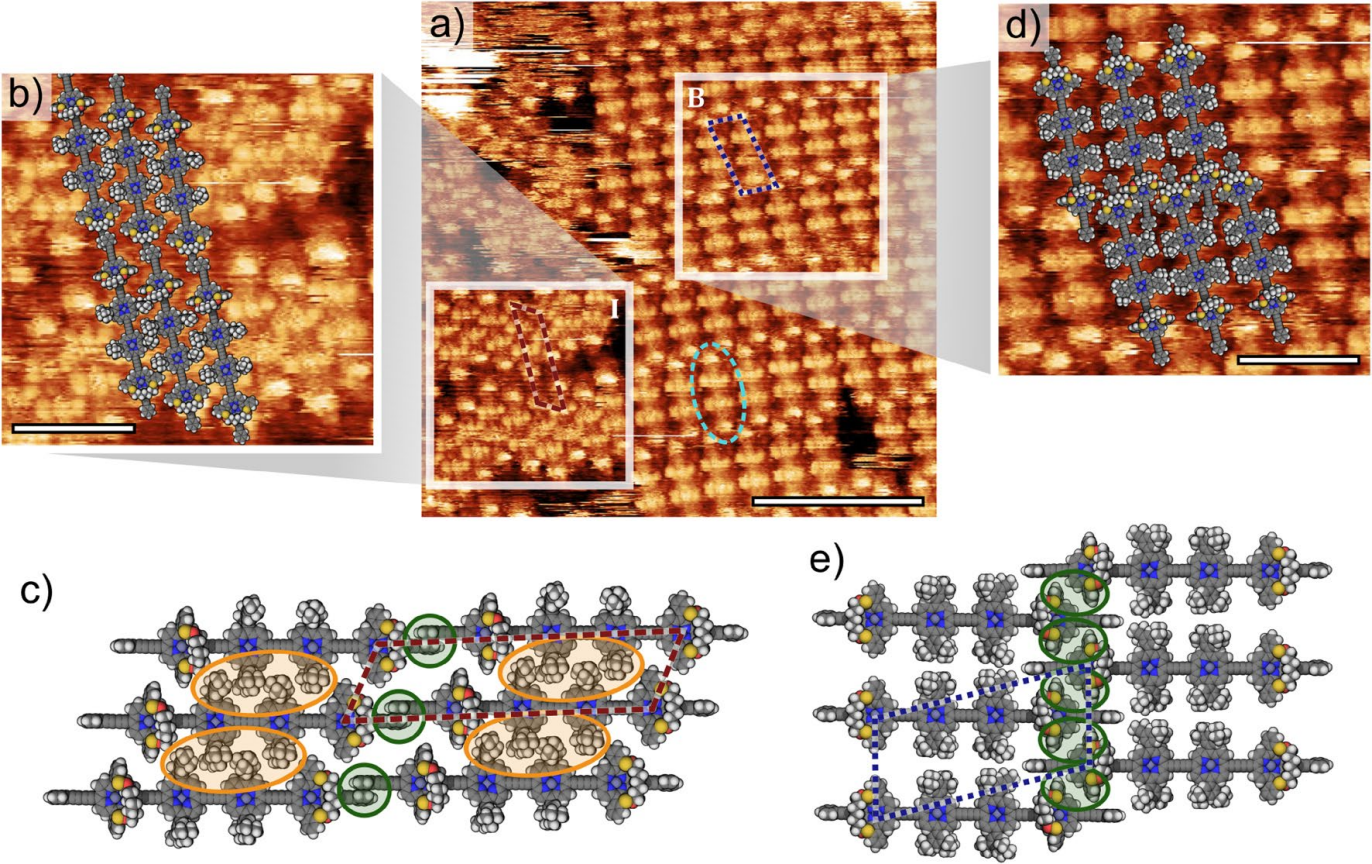
High resolution image for a 3.0 μM sample with: **(a)** bridge-stabilised structure (B) lattice marked with blue dotted line and interdigitated lattice (I) marked by magenta dashed line, **(b)** the interdigitated structure (I) with overlaid molecular model; **(c)** the interdigitated structure with phenyl-ring and *tert*-butylphenyl interactions highlighted in green and orange respectively; **(d)** the bridge-stabilised structure with overlaid molecular model; and **(e)** the bridge-stabilised structure with the bridge benzene ring interactions highlighted in green. Scale bars are **(a)** 1 nm, **(b)** 4 nm, and **(d)** 4 nm. Fitting carried out using LMAPper^30^.

Higher resolution STM images allow the sub-molecular structure of the oligomers to be interpreted (Fig. 2a). The ‘rods’ visible in Fig. 1b can now be resolved into four distinct features (see dashed ellipse in Fig. 2a). The two ‘brighter contrast’ features are assigned to the terminal porphyrin units (functionalised with thioester bridges), with the two central features assigned to di-*tert*-butyl phenyl (di-*t*BuPh) functionalised porphyrin units. The separation between the porphyrin units is found to be 1.34 ± 0.08 nm, in good agreement with our previous measurements for similar systems containing butadiyne linked porphyrins^11,18,29^.

It is clear from the STM image in Fig. 2a that the molecular structures within the two highlighted regions (white boxes) exhibit distinct packing motifs. We first consider the more densely-packed phase (labelled as I) shown in Fig. 2b: 0.119 ± 0.008 molecules nm^-2^. Based on a comparison between the spatially-resolved features in the STM images and a space-filling model based on the van der Waals radii of **S4-l-P4** it can be seen that the extended structures are stabilised by the interaction of the di-*t*BuPh groups in a similar manner to that observed for porphyrin polymer chains where the pendant groups were exclusively di-*t*BuPh units; see Fig. 2c (highlighted in orange)^18^. The unit cell for this interdigitated (I) structure is measured to be 6.0 ± 0.1 nm by 1.6 ± 0.1 nm (61 ± 1°) (indicated by the red dashed lines in Fig. 2a). The length of the molecule dictates the intra-row separation, and from a geometric consideration we suggest that the structure is additionally stabilised via a stacking interaction of the terminal phenyl rings (highlighted in green in Fig. 2c)^31^. The lack of contrast in the region between the rows is likely to be due to a lack of accessible electronic states compared to that present at the porphyrin units within the oligomer.

A lower density phase is observed to co-exist with the I-phase for the 3.0 μM preparation (indicated as B in Fig. 2a, with a close-up of the structure shown in Fig. 2d): 0.108 ± 0.007 molecules nm^-2^. This bridge-stabilised (B) structure is characterised with dimensions of 4.5 ± 0.2 nm by 2.1 ± 0.1 nm (78 ± 4°); blue dashed region in Fig. 2a. In this structure, the ordering appears to be driven by the interaction between the ‘end’ porphyrin units of the oligomer. As it is known the *meso*-linked benzene rings attached to the thioester bridges are rotated at ~ 70–90° relative to the core of the porphyrin^9,32,33^, we propose that an interaction between these two phenyl groups may stabilise the observed structures. We therefore conclude that the two close-packed phases are stabilised by distinct intermolecular interactions, with the interdigitating di-*t*BuPh groups stabilising the I (interdigitating) phase and the thioester groups of the terminal porphyrins stabilising the B (bridge-stabilised) phase.

**Figure 3:**
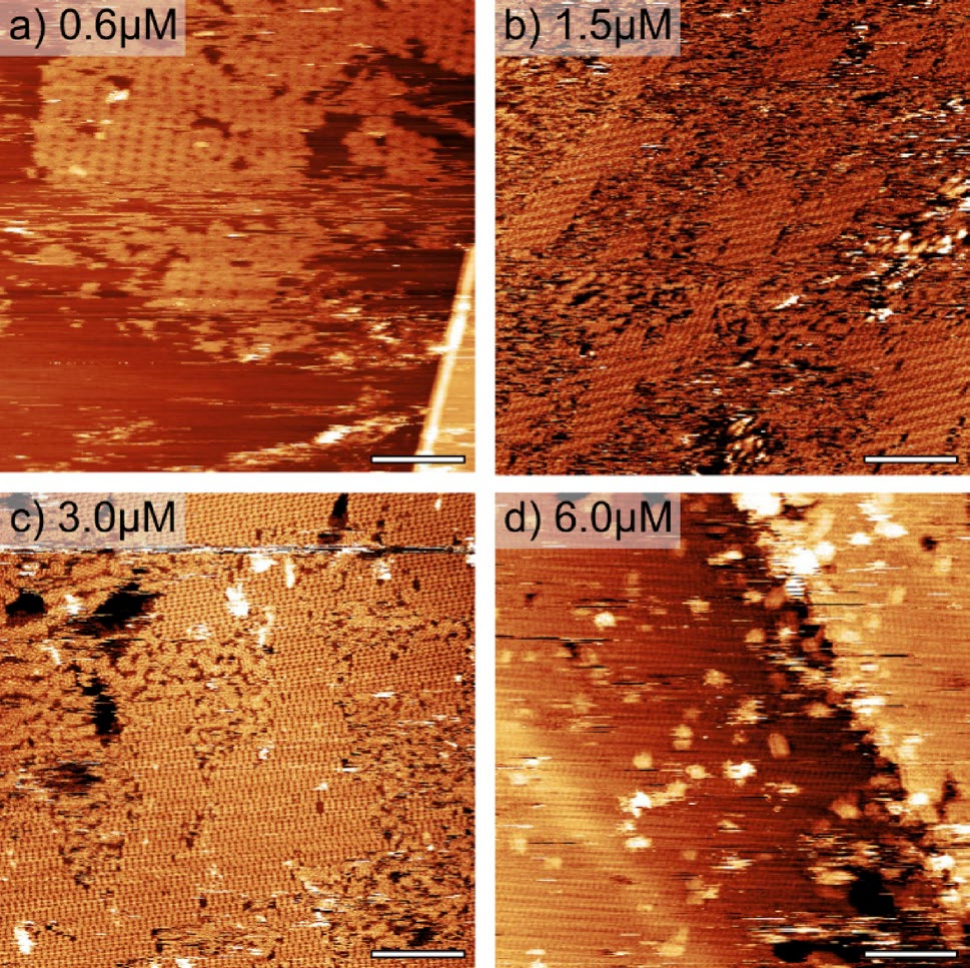
STM **i**mages obtained following deposition of solutions with four different concentrations; drop cast onto clean HOPG. Details of coverage and phases present are shown in Table 1. Scale bars 30 nm.

Following the structural characterisation of the two phases we now focus on the concentration-dependence of the structures formed. STM images were acquired for a range of concentrations of **S4-l-P4** dissolved in toluene and drop-cast onto HOPG. It is observed that the surface-coverage (as a fraction of a monolayer, ML) and the abundance of the two island types varies with solution concentration. Figure 3 shows STM images acquired following drop-cast deposition with 0.6, 1.5, 3.0, and 6.0 μM concentrations. We observe that, at intermediate concentrations a mix of B and I phases is observed, while increasing the concentration results in a high abundance of the B phase (0.97 ML of the material for 6.0 μM preparation). The surface coverage of the molecular islands, as a function of solution concentration, and the abundance of the B-phase for the four concentrations are shown in Table 1.

**Table 1:** Total coverage and abundance of the B phase for a range of concentrations.

Concentration (μM)	Coverage (ML)	B-Phase (ML)	Other (ML)
0.6	0.38	0.28	0.10
1.5	0.40	0.21	0.19
3.0	0.75	0.53	0.22
6.0	1.20	0.97	0.23

The data presented in Table 1 show that total coverage of the surface increases with concentration, with 0.38 ML coverage obtained for a concentration of 0.6 μM and 1.2 ML (i.e. 20% of the surface is covered with a second layer of molecules) at 6.0 μM. It is apparent that the B-phase is favoured at all concentrations, with a significant amount of **S4-l-P4** present within alternative structures in all cases. At low coverages (0.38 and 0.40 ML) 50–75% of the observed material is within B-phase islands, with the additional material existing within domains where the internal structure is not easily resolved; some small islands (~ 2% at 0.4 ML coverage) were identified as I-phase structures. Increasing the overall surface coverage to 0.75 ML results in an increase in the relative coverage of both the of B- and I-phases (0.53 ML and 0.22 ML, respectively), while at a coverage of 1.2 ML the amount of B-phase observed is further increased (0.97 ML) with no I -Phase identified at this coverage. Of note is the fact that higher total surface coverage does not lead to an increase in the amount of I-phase present, as would be expected if conversion from the lower density B-phase to the higher density I-Phase (increasing the overall density of molecules per surface area) was a significant factor underling the stability of the structures.

The prevalence of the B-phase for all surface coverages suggests that it is thermodynamically favoured under the conditions investigated, as compared to the I-phase. A simple interpretation of this observation is based upon the known conformational flexibility of porphyrin structures, specifically rotation of the di-*t*BuPh groups relative to the porphyrin core^34^, which has previously been shown to give rise to distinct packing motifs for porphyrin assemblies^35,36^. We propose that the two central porphyrins may exhibit two distinct conformations of the di-*t*BuPh groups, with respect to the porphyrin core, and that one of these conformations is present within each of the B- and I-phases. Within the B-phase both of the *tert*-butyl groups on each phenyl are able to be aligned such that they are brought towards the HOPG surface (see Fig. 2e, corresponding to a torsional angle of 20–30° between the porphyrin core and the di-*t*BuPh group). In contrast, within the I -phase only one of the two *tert*-butyl groups may be in contact with the underlying HOPG (see Fig. 2c, where the torsional angle is ~ 90^o^ in order to facilitate the interdigitation of the *tert*-butyl groups). These conformations are analogous to the ‘tilted’ and ‘upright’ states that were previously reported for di-*t*BuPh functionalised porphyrin oligomers^18^. The overall stability of the B-phase is therefore likely to be driven by a combination of molecule–molecule interactions (primarily an interaction between the thioester functionalised porphyrin units at the end of the oligomers) and the molecule–substrate interaction between the di-*t*BuPh groups and the HOPG surface.

For low surface coverage, where diffusion of **S4-l-P4** species away from the edges of the B islands is possible, the thermal energy at room temperature may facilitate conformational flexibility for the central two porphyrins, providing access to a meta-stable I-phase. When the surface coverage is ~ 1 ML, or greater, diffusion away from B islands is inhibited, and consequently the I-phase is not observed.

**Figure 4:**
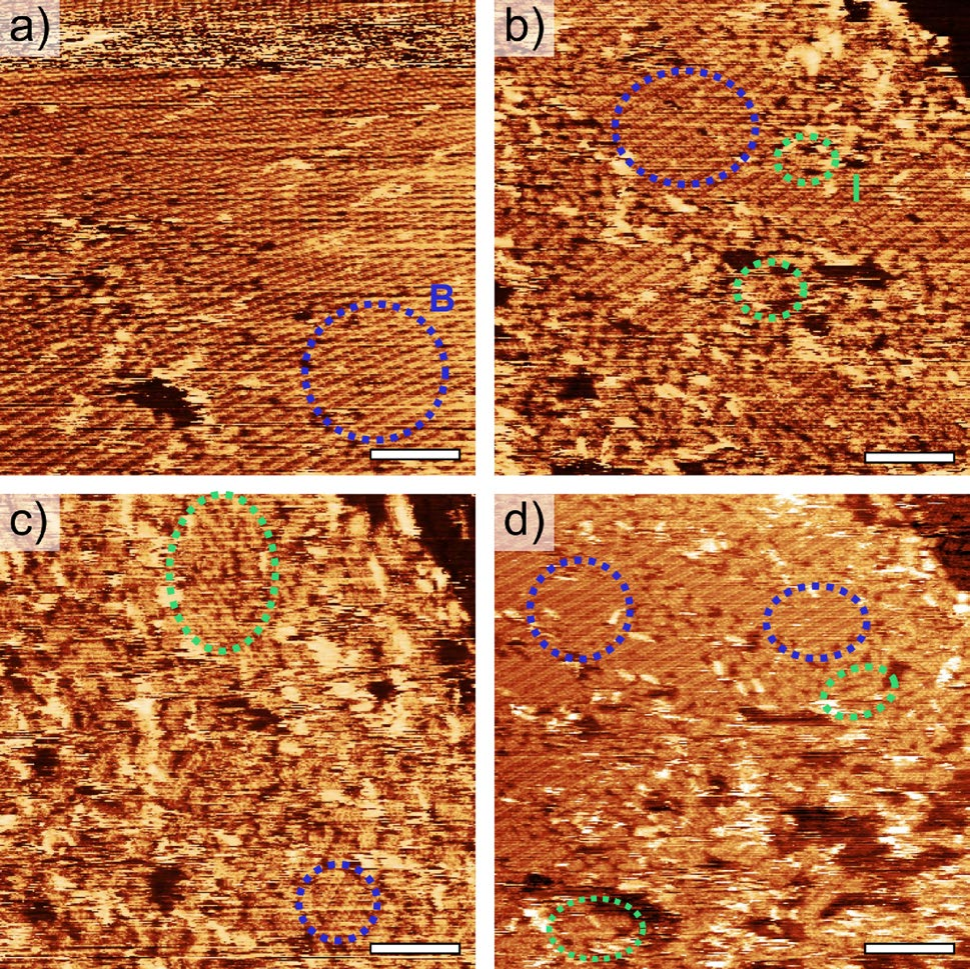
Structural change overserved over sequential images for a 3.0 μM sample showing change from large-scale ordered bridge-stabilised structure **(a)** to small islands of interdigitated structure **(c)**. Aligned using a step -edge to the upper right of the images. Each scan was acquired over 11 min, with total time between **(a)** and **(d)** of 67 min. Ellipses represent regions of bridged-stabilised (blue), and interdigitated (green) molecular structures.

The supposition that the I-phase is meta-stable is supported by observations of a reversible transition, B → I → B, following disruption of the surface by the STM probe. This is shown in Fig. 4, where following the preparation of a 3.0 μM sample a region of the B-phase is imaged. Figure 4a shows a large area of the B-phase; the non-linear appearance of the domain is due to thermal drift. Subsequent scanning of the same region (images are aligned using the step-edge feature indicated in Figs. 4b–d) results in the disruption of the highly ordered B-phase (Fig. 4b). Once disrupted, the molecules are observed, either to be mobile on the surface (such structures would be characterised as ‘other’ within the analysis presented in Table 1) or to form into small domains of the interdigitated structure. Further scanning resulted in the almost total loss of long-range order (Fig. 4c).

In Fig. 4b,c small regions of structure, not consistent with the dimensions of the B-phase, are observed as the STM probe was continuously scanned over a region for a period of 67 min. These structures are assigned to the I-phase, and are observed to spontaneously reform into the B-phase during the final scan, indicating that the I-phase is meta-stable on HOPG at room temperature.

In conclusion, we have shown that large, ordered arrays of strapped porphyrin oligomers can be formed via a simple solution deposition protocol. Due to the inherent complexity of the multiple functional groups, and the associated conformational flexibility, two different close-packed phases were observed. Based upon our STM characterisation of these phases, we suggest two distinct molecule–molecule interactions which drive the formation of the structures. By investigating the concentration dependence of these phases, we conclude that the bridge-stabilised (B) phase is a thermodynamically stable structure at room temperature. Our results demonstrate the potential for developing functional materials based upon the inherent properties of porphyrin oligomers, by utilising a facile solution-phase fabrication technique.

## Methods

**S4-l-P4** was synthesised as detailed in the SI. Molecular weight is 3323.49 g (C_206_H_170_N_16_O_4_S_4_Zn_4_). Solutions containing **S4-l-P4** were prepared by dissolving the material in toluene to produce a 50 μg/mL solution. Other concentrations were obtained via serial dilution. For sample preparation, a single droplet of the solution was deposited onto Scotch-tape-cleaved HOPG and left to dry in air. Following deposition, samples were transferred to an STM [Molecular Imaging (Agilent) PicoSTM] and a 10 μL droplet of nonanoic acid was deposited for imaging. Pt/Ir (80%:20%) wire was cut and used as the STM probe. Samples were imaged in constant current mode with a bias of 1.0 V applied to the tip and a tunnelling current of 0.05 nA. Additional details of sample preparation and image analysis may be found in the SI.

## Supplementary Information

Supplementary Information.

## Data Availability

Supplementary information available on: Additional STM data showing extended ordered arrays of **S4-l-P4** on HOPG; Details of the molecular models; Details of tip-induced disruption of molecular structures; Details of the synthesis and characterisation of **S4-l-P4**; This material is available free of charge via the internet at: https://www.nature.com/srep/. The experimental data on which this work is based is available at https://doi.org/10.17639/nott.7148.
