# Supplementary material for: Self-assembly of a strapped linear porphyrin oligomer on HOPG
Source: Sci Rep. 2021 Oct 14;11:20388. doi: 10.1038/s41598-021-99881-x (PMC8516934; doi:10.1038/s41598-021-99881-x)
Supplement: Supplementary file 1 — Supplementary Information. [file 41598_2021_99881_MOESM1_ESM.pdf]

Self-assembly of a strapped linear porphyrin oligomer on HOPG

Abigail Bellamy-Carter[1], Cécile Roche[2], Harry L. Anderson[2], Alex Saywell[1*]

[1]School of Physics & Astronomy, University of Nottingham, Nottingham, NG7 2RD, UK.

[2]Department of Chemistry, University of Oxford, Oxford OX1 3TA, United Kingdom

*Corresponding author A.S. (alex.saywell@nottingham.ac.uk)

Contents

Additional Experimental Data S2
LMAPper Method S2
Image Alignment Method S3
Synthesis and Characterisation of **S4-*l*-P4** S4

Additional Experimental Data

Images showing that the bridge-stabilised structure forms extended ordered arrays.

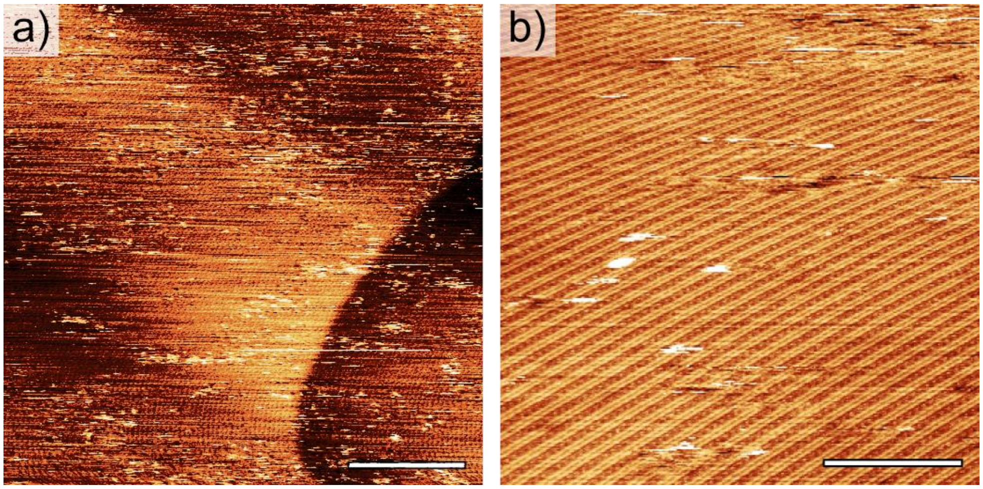

Figure S1 | Overview scans for (a) 6.0 µM and (b) 3.0 µM samples, showing extended arrays of the ordered structures. Scale bars are (a) 50 nm and (b) 20 nm.

LMAPper Method

Structural models were created by loading a molecular model of S4-*l*-P4, created in MarvinSketch and edited in Pymol, into LMAPper alongside the STM image of the surface (scaling information was provided for both imported files). The skeletal molecules were then arranged to best fit the image, based upon known bonding motifs and the van der Waals radii of the molecules (see Figure S2). The models produced within LMAPper were used to produce the structure models shown within Figure 2 of the main manuscript.

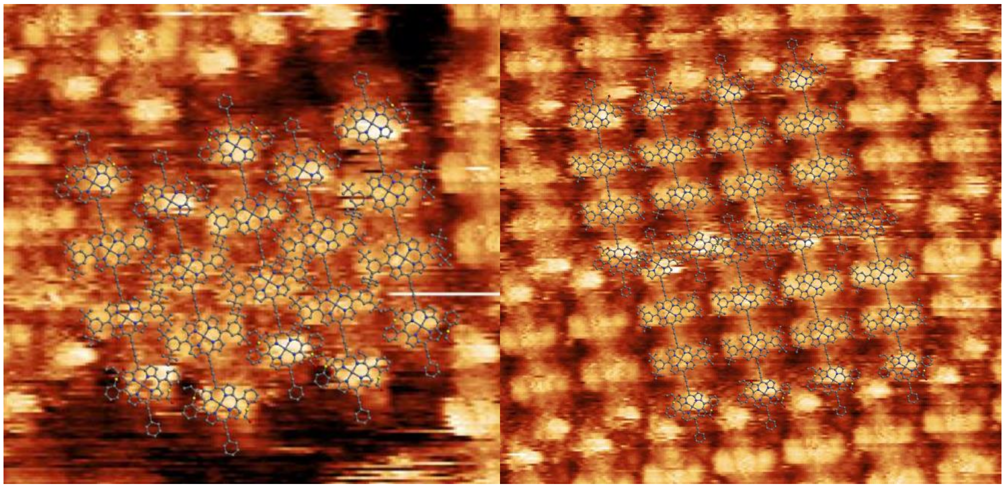

Figure S2 | Images from LMAPper used to create the models in Figure 2: (left) the interdigitated structure and (right) the bridge-stabilised structure.

Image Alignment Method

The images presented in Figure 4 were acquired sequentially over the course of 67 minutes (with each scan taking approximately 11 minutes). The images for (b)-(d) were aligned utilising a step-edge, and features along it, to orientate the scans relative to each other.

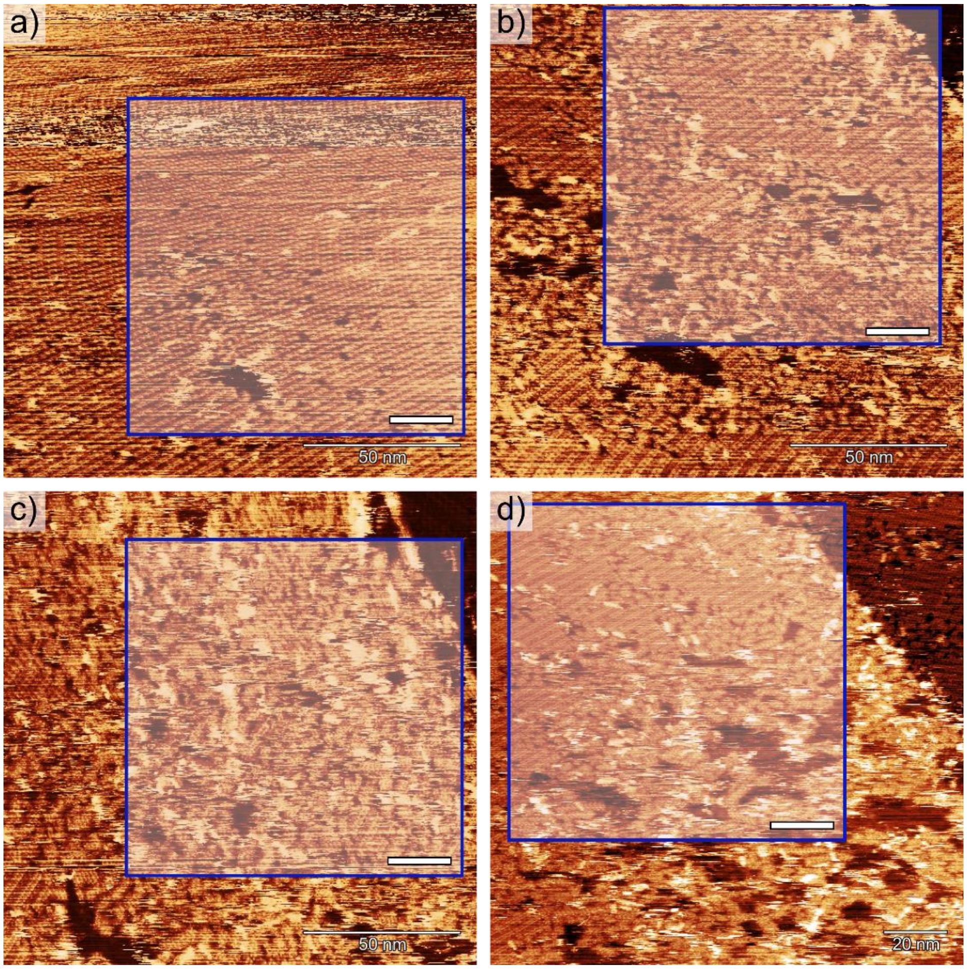

Figure S3 | STM images which form the basis for Figure 3. Blue boxes show the area selected for inclusion prior to alignment of the step-edge feature.

Synthesis and Characterisation of S4-*l*-P4

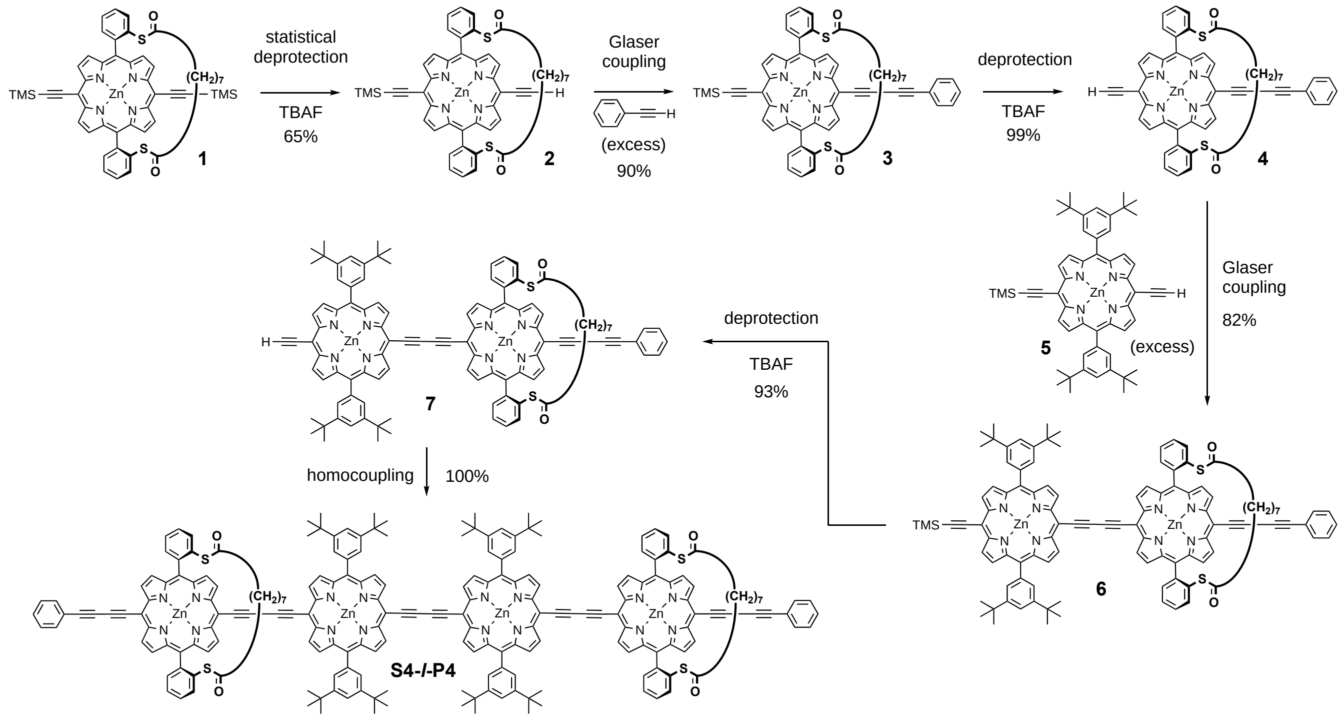

Scheme S1. The synthetic route used for the preparation of **S4-*l*-P4**.

Starting materials and instrumentation. All reagents were purchased from commercial sources and solvents were use as supplied unless otherwise noted. The starting materials **1** and **5** were synthesized following published procedures [C. Roche, Q. Luo, G. Gil-Ramírez, H.-W. Jiang, D. R. Kohn, Y. Xiong, A. L. Thompson, H. L. Anderson, *J. Org. Chem.* **2017**, *82*, 7446−7462; P. N. Taylor, A. P. Wylie, J. Huuskonen, H. L. Anderson, *Angew. Chem. Int. Ed.* **1998**, *37*, 986–989]. Dry solvents (THF, $CHCl_3$, CH_2Cl_2 and toluene) were obtained by passing through alumina under N_2. Diisopropylamine was dried over calcium hydride, distilled and stored under N_2 over molecular sieves. NMR data were collected at 400 MHz or at 500 MHz at 298 K. Chemical shifts are quoted as parts per million (ppm) relative to residual $CHCl_3$ (at δ 7.26 ppm for ^{1}H NMR and at δ 77.16 ppm for ^{13}C NMR), and coupling constants (J) are reported in Hertz. Size exclusion chromatography (SEC) was carried out using Bio-Beads S-X1, 200–400 mesh (Bio Rad).

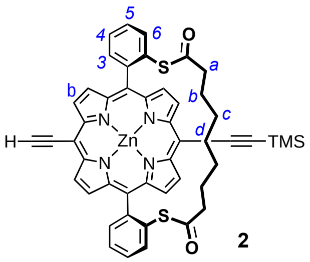

Mono-deprotected C7-strapped porphyrin 2. TMS-protected strapped porphyrin monomer **1** (40 mg, 39 µmol) was dissolved in $CHCl_3$ (20 mL), and tetra-*n*-butylammonium fluoride (TBAF) (20 µL, 1.0 M solution in THF, 20 µmol) was added. After stirring at 20 °C for 40 min the reaction mixture was passed through a short plug of silica gel (CH_2Cl_2/pyridine 100:1). After evaporation of the solvents, the starting material, mono-deprotected product **2**, and bis-deprotected product were separated by flash column chromatography on silica using a gradient of eluents from cyclohexane/ethyl acetate/pyridine 25:1:1 to 5:1:1. The same procedure was repeated twice on the recovered starting material from each batch. The fractions of mono-deprotected product were combined to give **2** as its 1:1 complex with pyridine (purple solid, 24 mg, 65%). ^{1}H NMR ($CDCl_3$, 400 MHz): δ = 9.56 (d, ^3J = 4.4 Hz, 2H, H_β), 9.55 (d, ^3J = 4.4 Hz, 2H, H_β), 8.59 (overlapping d, ^3J = 4.4 Hz, 4H, H_β), 8.48 (m, 2H, H_3), 7.89–7.82 (m, 6H, $H_{4,5,6}$), 6.37 (m, 1H, H_{py}), 5.59 (m, 2H, H_{py}), 4.09 (s, 1H, alkyne-*H*), 2.91 (m, 2H, H_{py}), 1.34 (m, 4H, $(CH_2)_a$), 0.57 (s, 9H, -Si*Me*$_3$), –1.05 (m, 8H, $(CH_2)_{b,c}$), –2.05 (m, 1H, $(CH_2)_d$), –2.18 ppm (m, 1H, $(CH_2)_d$). ^{13}C NMR ($CDCl_3$, 100 MHz): δ = 196.81, 152.55, 152.40, 150.32, 147.15, 143.59, 136.82, 136.06,

134.01, 132.43, 132.22, 132.11, 131.42, 131.24, 129.26, 128.13, 122.49, 120.41, 108.25, 101.22, 99.56, 86.68, 83.38, 42.94, 26.96, 25.96, 25.75, 0.53 ppm.

Phenyl-protected C7-strapped porphyrin 3. Mono-deprotected C7-strapped porphyrin **2** (21 mg, 22 μmol) was dissolved in dry toluene (8 mL) and distilled *i*-Pr_2NH (0.5 mL), and phenylacetylene (29 μL, 0.27 mmol) was added by syringe. $Pd(PPh_3)_2Cl_2$ (6.1 mg, 8.7 μmol), CuI (17 mg, 87 μmol), and 1,4-benzoquinone (38 mg, 0.35 mmol) were dissolved in dry toluene (7 mL) and distilled *i*-Pr_2NH (1.5 mL), and the resulting solution was added to the porphyrin solution. The reaction mixture was stirred at 20 °C for 1 h and then passed through a short silica gel column using CH_2Cl_2 with 1% pyridine. The product was purified by flash silica column using a gradient of eluents from cyclohexane/EtOAc/pyridine 25:1:1 to 5:1:1, to give compound **3** as its 1:1 complex with pyridine (purple solid, 21 mg, 90%). ^{1}H NMR ($CDCl_3$, 400 MHz): δ = 9.54 (d, ^3J = 4.5 Hz, 2H, H_β), 9.53 (d, ^3J = 4.5 Hz, 2H, H_β), 8.59 (d, ^3J = 4.5 Hz, 2H, H_β), 8.56 (d, ^3J = 4.5 Hz, 2H, H_β), 8.47 (m, 2H, H_3), 7.89–7.85 (m, 6H, $H_{4,5,6}$), 7.72 (m, 2H, H_{Ph}), 7.43 (m, 3H, H_{Ph}), 6.50 (m, 1H, H_{py}), 5.74 (m, 2H, H_{py}), 3.34 (m, 2H, H_{py}), 1.37 (m, 4H, $(CH_2)_a$), 0.57 (s, 9H, -Si*Me*$_3$), –1.01 (m, 8H, $(CH_2)_{b,c}$), –2.00 (m, 1H, $(CH_2)_d$), –2.18 ppm (m, 1H, $(CH_2)_d$). ^{13}C NMR ($CDCl_3$, 100 MHz): δ = 196.85, 153.12, 152.33, 150.43, 150.00, 147.00, 144.01, 136.85, 136.18, 133.95, 132.69, 132.18, 131.98, 131.48, 130.94, 129.31, 129.27, 128.67, 128.61, 128.16, 122.67, 122.41, 120.82, 108.13, 101.79, 101.48, 99.04, 85.65, 83.87, 80.49, 75.59, 42.97, 27.05, 26.00, 25.82, 0.51 ppm.

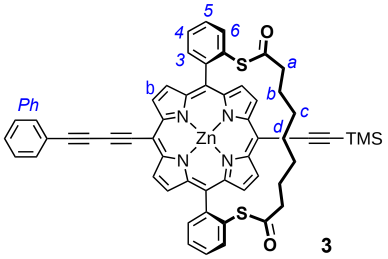

3

Deprotected C7-strapped porphyrin monomer 4. TMS-protected strapped porphyrin monomer **3** (19 mg, 18 μmol) was dissolved in CH_2Cl_2 (7 mL), and tetra-*n*-butylammonium fluoride (TBAF) (18 μL, 1.0 M solution in THF, 18 μmol) was added. After stirring at 20 °C for 10 min the reaction mixture was passed through a short plug of silica gel (CH_2Cl_2/pyridine 100:1). Evaporation of the solvents gave deprotected compound **4** as its 1:1 complex with pyridine (purple solid, 18 mg, 99%). ^{1}H NMR ($CDCl_3$, 400 MHz): δ = 9.55 (d, ^3J = 4.5 Hz, 2H, H_β), 9.55 (d, ^3J = 4.5 Hz, 2H, H_β), 8.59 (d, ^3J = 4.5 Hz, 2H, H_β), 8.57 (d, ^3J = 4.5 Hz, 2H, H_β), 8.48 (m, 2H, H_3), 7.89–7.82 (m, 6H, $H_{4,5,6}$), 7.72 (m, 2H, H_{Ph}), 7.43 (m, 3H, H_{Ph}), 6.43 (m, 1H, H_{py}), 5.65 (m, 2H, H_{py}), 4.11 (s, 1H, alkyne-*H*), 3.05 (m, 2H, H_{py}), 1.35 (m, 4H, $(CH_2)_a$), –0.93 (m, 8H, $(CH_2)_{b,c}$), –1.99 ppm (m, 2H, $(CH_2)_d$). ^{13}C NMR ($CDCl_3$, 100 MHz): δ = 196.64, 153.14, 152.50, 150.56, 150.13, 146.95, 143.71, 136.89, 136.15, 133.88, 132.73, 132.70, 132.42, 132.08, 131.43, 131.07, 129.34, 129.29, 128.69, 128.15, 122.56, 122.45, 120.85, 100.40, 99.31, 86.54, 85.56, 83.97, 83.71, 80.66, 75.60, 42.99, 27.22, 26.18, 25.83 ppm.

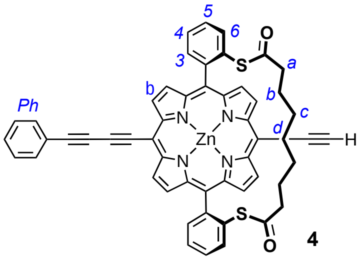

4

C7-strapped porphyrin dimer 6. Deprotected C7-strapped porphyrin monomer **4** (15.5 mg, 16.0 μmol) and mono-deprotected *t*Bu porphyrin monomer **5** (182 mg, 192 μmol) were dissolved in dry toluene (15 mL) and distilled *i*-Pr_2NH (0.5 mL). $Pd(PPh_3)_2Cl_2$ (4.4 mg, 6.2 μmol), CuI (12 mg, 62 μmol) and 1,4-benzoquinone (27 mg, 0.25 mmol) were dissolved in dry toluene (5 mL) and distilled *i*-Pr_2NH (1.5 mL), and the catalyst solution was added to the porphyrin solution. The reaction mixture was stirred at 20 °C for 3 h and passed through a short silica gel column using CH_2Cl_2 with 1% pyridine. The mixture of products was purified by flash silica column using a gradient of eluents from cyclohexane/EtOAc/pyridine 25:1:1 (elution of the dimer of the *t*Bu porphyrin) to 8:1:1 (elution of the desired dimer **6**). Porphyrin dimer **6** (25 mg, 82%) was isolated as a dark green/brown solid (1:2 complex with pyridine). ^{1}H NMR ($CDCl_3$, 400 MHz): δ = 9.91 (d, ^3J = 4.5 Hz, 2H, H_β), 9.80 (d, ^3J = 4.5 Hz, 2H, H_β), 9.67 (d, ^3J = 4.5 Hz, 2H, H_β), 9.55 (d, ^3J = 4.5 Hz, 2H, H_β), 9.02 (d, ^3J = 4.5 Hz, 2H, H_β), 8.92 (d, ^3J = 4.5 Hz, 2H,

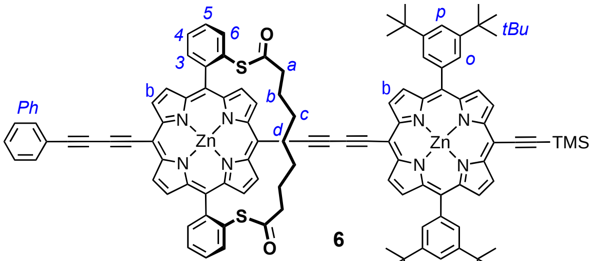

6

H_β), 8.66 (d, ^3J = 4.5 Hz, 2H, H_β), 8.59 (d, ^3J = 4.5 Hz, 2H, H_β), 8.53 (m, 2H, H_3), 8.08 (d, ^4J = 1.5 Hz, 4H, Ar-H_o), 7.93–7.84 (m, 6H, $H_{4,5,6}$), 7.84 (m, 2H, Ar-H_p), 7.75 (m, 2H, H_{Ph}), 7.44 (m, 3H, H_{Ph}), 6.46 (m, 2H, H_{py}), 5.68 (m, 4H, H_{py}), 2.96 (m, 4H, H_{py}), 1.59 (s, 36H, *t*Bu), 1.42 (m, 4H, $(CH_2)_a$), 0.63 (s, 9H, *TMS*), –0.83 (m, 8H, $(CH_2)_{b,c}$), –1.85 ppm (m, 2H, $(CH_2)_d$). ^{13}C NMR ($CDCl_3$, 100 MHz): δ = 196.71, 153.21, 153.05, 152.20, 150.91, 150.44, 150.26, 148.74, 146.92, 143.62, 141.73, 136.94, 136.20, 133.88, 133.43, 132.96, 132.75, 132.70, 132.07, 131.76, 131.33, 131.10, 131.01, 130.70, 130.13, 129.39, 129.32, 128.71, 128.28, 128.21, 128.16, 124.61, 122.63, 122.45, 121.38, 121.02, 108.62, 101.39, 101.34, 101.05, 99.80, 99.23, 89.17, 88.09, 85.58, 84.12, 82.97, 82.34, 80.88, 75.65, 43.04, 35.22, 31.95, 27.39, 26.28, 25.94, 0.58 ppm.

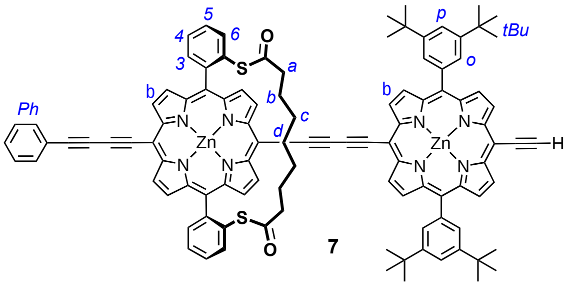

Deprotected C7-strapped porphyrin dimer 7. TMS-protected porphyrin dimer **6** (23 mg, 12 µmol) was dissolved in CH_2Cl_2 (5 mL), and tetra-*n*-butylammonium fluoride (TBAF) (12 µL, 1.0 M solution in THF, 12 µmol) was added. After stirring at 20 °C for 30 min the reaction mixture was passed through a short plug of silica gel (CH_2Cl_2/pyridine 100:1). Evaporation of the solvents gave deprotected compound **7** as its 1:2 complex with pyridine (brown solid, 21 mg, 93%). ^{1}H NMR ($CDCl_3$/pyridine-d_5 1%, 400 MHz): δ = 9.91 (d, ^3J = 4.5 Hz, 2H, H_β), 9.80 (d, ^3J = 4.5 Hz, 2H, H_β), 9.68 (d, ^3J = 4.5 Hz, 2H, H_β), 9.55 (d, ^3J = 4.5 Hz, 2H, H_β), 9.01 (d, ^3J = 4.5 Hz, 2H, H_β), 8.93 (d, ^3J = 4.5 Hz, 2H, H_β), 8.67 (d, ^3J = 4.5 Hz, 2H, H_β), 8.61 (d, ^3J = 4.5 Hz, 2H, H_β), 8.53 (m, 2H, H_3), 8.07 (d, ^4J = 1.4 Hz, 4H, Ar-H_o), 7.93–7.86 (m, 6H, $H_{4,5,6}$), 7.84 (m, 2H, Ar-H_p), 7.74 (m, 2H, H_{Ph}), 7.44 (m, 3H, H_{Ph}), 7.13 (m, 2H, H_{py}), 6.57 (m, 4H, H_{py}), 6.14 (m, 4H, H_{py}), 4.17 (s, 1H, alkyne-*H*), 1.59 (s, 36H, *t*Bu), 1.44 (m, 4H, $(CH_2)_a$), –0.82 (m, 8H, $(CH_2)_{b,c}$), –1.85 ppm (m, 2H, $(CH_2)_d$). ^{13}C NMR ($CDCl_3$/pyridine-d_5, 100 MHz): δ = 196.68, 153.22, 153.02, 152.29, 150.96, 150.53, 150.30, 148.74, 147.20, 146.95, 146.72, 146.45, 141.73, 136.96, 136.11, 133.91, 133.46, 133.12, 132.73, 132.72, 132.10, 131.32, 131.10, 130.93, 130.75, 130.14, 129.39, 129.31, 128.70, 128.20, 124.56, 123.31, 123.01, 122.76, 122.51, 122.45, 121.41, 121.03, 100.98, 99.89, 99.78, 99.31, 89.12, 88.13, 87.01, 85.60, 84.11, 83.55, 82.91, 82.35, 80.85, 75.61, 43.08, 35.22, 31.94, 27.35, 26.27, 25.94 ppm.

C7-strapped porphyrin tetramer S4-*l*-P4.

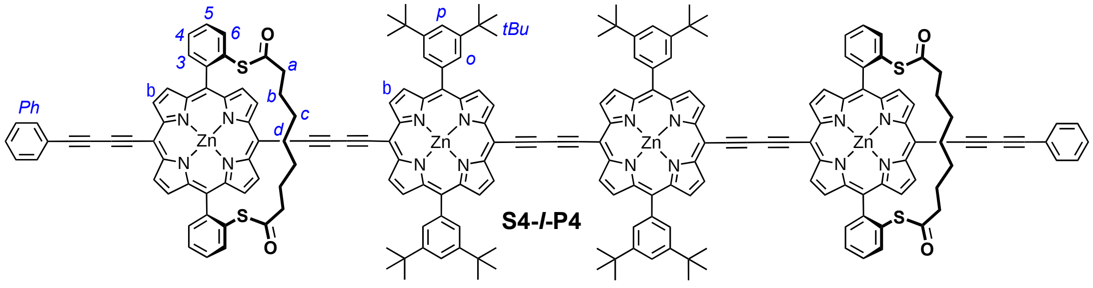

Deprotected C7-strapped porphyrin dimer **7** (18 mg, 9.8 µmol) was dissolved in dry toluene (1.5 mL) and distilled *i*-Pr_2NH (50 µL). $Pd(PPh_3)_2Cl_2$ (0.21 mg, 0.29 µmol), CuI (0.56 mg, 2.9 µmol) and 1,4-benzoquinone (1.3 mg, 12 µmol) were dissolved in dry toluene (0.5 mL) and distilled *i*-Pr_2NH (0.15 mL), and the catalyst solution was added to the porphyrin solution. The reaction mixture was stirred at 20 °C for 3 h and passed through a short silica gel column using CH_2Cl_2 with 1% pyridine. The product was further purified on a Biobeads SX1 size-exclusion column in toluene/pyridine 1%, to give porphyrin tetramer **S4-*l*-P4** (18 mg, 100%) as a brown solid (1:4 complex with pyridine). ^{1}H NMR ($CDCl_3$, 400 MHz): δ = 9.94 (d, ^3J = 4.5 Hz, 4H, H_β), 9.92 (d, ^3J = 4.5 Hz, 4H, H_β), 9.82 (d, ^3J = 4.5 Hz, 4H, H_β), 9.56 (d, ^3J = 4.5 Hz, 4H, H_β), 9.03 (overlapping d, ^3J = 4.5 Hz, 8H, H_β), 8.69 (d, ^3J = 4.5 Hz, 4H, H_β), 8.61 (d, ^3J = 4.5 Hz, 4H, H_β), 8.54 (m, 4H, H_3), 8.13 (d, ^4J = 1.4 Hz, 8H, Ar-H_o), 7.94–7.87 (m, 12H, $H_{4,5,6}$), 7.87 (m, 4H, Ar-H_p), 7.75 (m, 4H, H_{Ph}), 7.45 (m, 3H, H_{Ph}), 6.53 (m,

4H, H_{py}), 5.77 (m, 8H, Hpy), 3.18 (m, 8H, H_{py}), 1.62 (s, 72H, *t*Bu), 1.44 (m, 8H, $(CH_2)_a$), –0.81 (m, 16H, $(CH_2)_{b,c}$), –1.84 ppm (m, 4H, $(CH_2)_d$). ^{13}C NMR ($CDCl_3$, 100 MHz): δ = 196.70, 153.24, 153.08, 150.72, 150.31, 148.85, 146.93, 143.85, 141.66, 136.98, 136.28, 133.91, 133.51, 133.47, 132.76, 132.25, 132.11, 131.34, 131.14, 130.86, 130.22, 129.42, 129.34, 129.18, 128.72, 128.37, 128.24, 127.73, 125.44, 125.21, 122.75, 122.45, 121.45, 121.14, 100.96, 100.36, 100.02, 99.87, 89.11, 88.96, 88.36, 85.57, 84.15, 82.95, 82.91, 82.68, 80.91, 75.63, 43.08, 35.27, 31.98, 27.39, 26.29, 25.95 ppm.

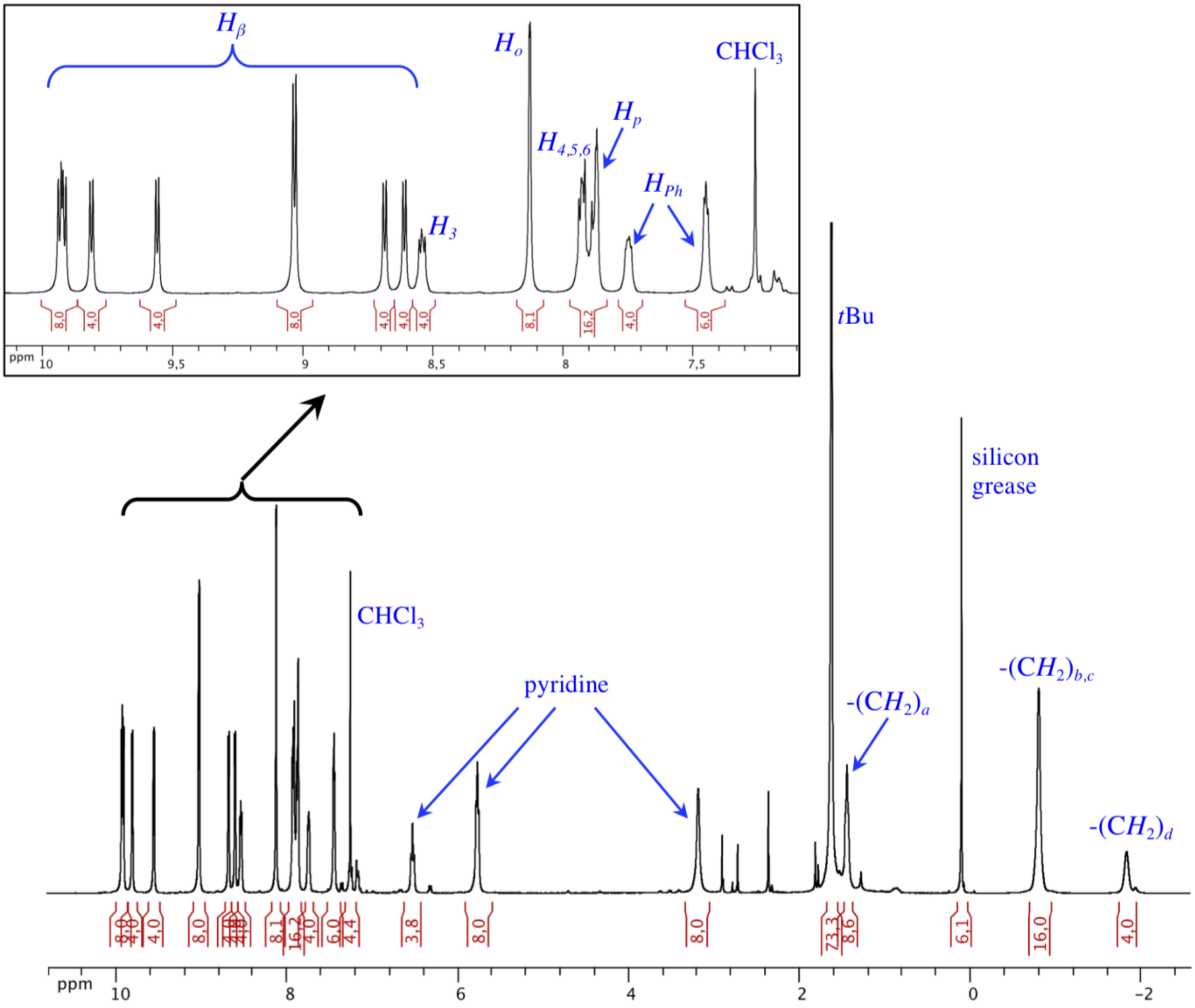

Figure S4 | ^{1}H NMR spectrum of porphyrin tetramer **S4-*l*-P4** with 4 equivalents of coordinated pyridine ($CDCl_3$, 400 MHz).
